# Elevated glutamine but not glutamate is associated with clozapine eligibility in an early psychosis sample

**DOI:** 10.3389/fpsyt.2026.1762696

**Published:** 2026-02-24

**Authors:** Maxwell Seward, Esther Puiras, Temi Toba-Oluboka, Candice E. Crocker, Philip G. Tibbo, Kara Dempster

**Affiliations:** 1 Department of Psychiatry, Dalhousie University, Halifax, NS, Canada; 2 Nova Scotia Health Authority, Halifax, NS, Canada; 3 Department of Diagnostic Radiology, Dalhousie University, Halifax, NS, Canada

**Keywords:** anterior cingulate cortex, clozapine eligibility, early phase psychosis, glutamate, glutamine, magnetic resonance spectroscopy, treatment resistant schizophrenia

## Abstract

**Introduction:**

Approximately one-third of individuals with schizophrenia will meet the criteria for treatment-resistant schizophrenia (TRS), the majority of whom demonstrate poor pharmacological response early in their course of illness. Clozapine response is higher when used early in the illness trajectory; thus, there is a need to characterize the neurobiological underpinnings of TRS to support early stratification to clozapine. We hypothesized that elevated glutamate in the anterior cingulate cortex (ACC), measured using *in vivo* magnetic resonance spectroscopy (MRS), would be associated with clozapine eligibility in an early-phase psychosis (EPP) cohort.

**Methods:**

Criteria-defined clozapine-eligible (CE) individuals and treatment responders (TR) with non-affective EPP were recruited from the Nova Scotia Early Psychosis Program (within the first 5 years of illness onset). Clinical assessments were completed as well as 3T ^1^H-MRS to measure glutamate in the bilateral dorsal ACC. ^1^H-MRS acquisitions were performed using Point RESolved Spectroscopy (TE = 40 ms, TR = 2,000 ms; 128 averages).

**Results:**

Forty-six EPP individuals completed the study, with 24 meeting the criteria for clozapine eligibility (mean age = 24) and 22 as treatment responders (mean age = 22). Twenty-six individuals (56.5%) in the total sample were receiving treatment with a long-acting injectable antipsychotic (LAI) medication. The TR group (16 men, 6 women) did not differ from the CE group (19 men, 5 women) in age, years of education, family history of psychosis, or regular nicotine and cannabis use. The CE group had higher PANSS scores, a longer duration of untreated psychosis, and worse social functioning and were taking a higher burden of antipsychotic treatment [chlorpromazine (cpz) equivalencies]. Clinical variables that were significantly different between groups were added to the linear model, and nested model comparisons were used to select the final model for analysis of each metabolite. Glutamate was compared between the groups using a one-way ANOVA, and glutamine was compared using ANCOVA. While ACC glutamate was not found to be significantly different between the groups, glutamine, a precursor for glutamate, was higher in the CE group (*M* = 4.43, SD = 0.73) relative to the TR group (*M* = 4.02, SD = 0.64) [*F* (1, 40) = 5.44, *p* = 0.02].

**Discussion:**

While elevated ACC glutamate has been associated with poor response to antipsychotic medications in early psychosis samples, this is the first study to explore the association with clozapine eligibility. Contrary to our hypothesis, ACC glutamate was not higher in the CE group. However, glutamine, a precursor to glutamate, was higher in the CE group, in line with previous studies that have found elevated glutamatergic metabolites to be associated with poor treatment response to antipsychotic medication. Our results support future studies to further characterize the neurobiology of clozapine eligibility in early-phase psychosis to assist in the timely initiation of clozapine to maximize outcomes.

## Background

Approximately one-third of individuals with schizophrenia do not respond to first-line antipsychotic medications and meet the criteria for treatment-resistant schizophrenia (TRS) (1). TRS can be identified early in the course of illness, with 70%–84% of those with TRS being poor responders to antipsychotic medication from their first episode of psychosis (2, 3). Considerable evidence supports the use of clozapine for TRS (4). Timely identification of individuals who are clozapine-eligible is of prime importance within early psychosis care, as the response to clozapine is more robust when used earlier in the illness trajectory (5, 6).

In clinical practice, clozapine eligibility (CE)—defined as non-response to two adequate trials of first-line antipsychotics—represents the standard operational criterion used to identify individuals with probable TRS and to guide treatment escalation. CE is synonymous with TRS, and it provides a clinically meaningful proxy reflective of repeated treatment failures. The requirement that individuals fail two antipsychotic trials before meeting CE does not account for accumulating evidence that those who ultimately benefit from clozapine may possess distinct neurobiological profiles when compared to treatment responders. Accordingly, a uniform trial-and-error approach to treatment may contribute to prolonged periods of active psychosis prior to clozapine initiation, leading to both prolonged individual opportunity loss and healthcare burden. Therefore, the ability to prognosticate clozapine eligibility based on objective neurological findings represents an important opportunity toward earlier identification of TRS and more timely intervention.

First-line antipsychotic medications reduce psychotic symptoms primarily through dopaminergic blockade (7, 8). TRS patients do not respond to dopamine-blocking agents, further supporting the theory of alternative neurobiological profiles in this subset of individuals (9–11). In fact, it has been demonstrated that responders to antipsychotic treatment have higher dopamine synthesis capacity, while those who do not respond have normal dopamine synthesis capacity but higher levels of glutamate in the anterior cingulate cortex (ACC) (9). The ACC is the medial frontal brain region that is part of the salience network (12), a brain network responsible for switching between focus on self and task-relevant, directed attention on external stimuli (13). An aberrant salience hypothesis of schizophrenia has been proposed (14). In this model, dysregulation of the salience network leads to inappropriate assignment of salience to elements of an individual’s perception (14), resulting in psychotic symptoms. Functional magnetic resonance imaging (fMRI) studies have consistently implicated atypical ACC activity in schizophrenia (15), and this atypical activity has been associated with more severe symptoms, including delusions (16) and negative symptoms such as disorganization (17) or apathy (18). The salience network and ACC are also specifically dysregulated in TRS. ^1^H-MRS studies have shown that ACC glutamate is elevated in TRS populations relative to treatment-responding populations (9, 19, 20). Ochi and colleagues (21) found that reductions in cortical thickness and increased mean diffusivity on the surface of the cingulate sulcus were associated with elevated glutamate metabolites in the ACC of individuals with TRS. This suggests that excess glutamate seen in TRS may lead to structural alterations in subdivisions of the ACC associated with inhibitory control. Relatedly, Matrone and colleagues (22) found that in TRS, reductions in fractional anisotropy in several white matter tracts were correlated with impaired cognitive performance and more severe symptoms. Furthermore, elevated ACC glutamate in TRS was associated with worse verbal memory. Based on their results, they suggested a model of glutamatergic excitotoxicity, leading to neuronal damage and dysconnectivity in the PFC.

Expanding on evidence linking ACC glutamate and TRS, prior work has investigated the role of ACC glutamate in treatment response from the very early stages of illness. In early-phase psychosis (EPP), higher ACC glutamate has been demonstrated in patients who do not achieve remission within a discrete time period, for example, after 6 months (23). Similarly, elevated ACC glutamate at baseline was associated with lack of remission following 1 month (24), 6 weeks (25), and 3 months (26) of antipsychotic treatment. However, others have failed to find similar associations between ACC glutamate and response to treatment (27–29). This includes one study (30) which found that *lower* ACC glutamate at baseline was associated with non-remission after 8 weeks of risperidone treatment, although the sample of non-remitted patients was quite small (*N* = 10). Additionally, van der Pluijm and colleagues (31) failed to replicate the association of higher baseline ACC glutamate in non-responders to antipsychotic medication after 6 months of antipsychotic treatment using a response threshold more closely related to clinical standards of non-response (e.g., ongoing psychotic symptoms despite two antipsychotic trials). While early response patterns are associated with longitudinal outcomes, failure to respond to medication within a discrete time is not necessarily associated with meeting criteria for clozapine eligibility over time, a more nuanced but related clinical phenomenon.

## The present study

As clozapine is the gold standard treatment for TRS, clozapine eligibility and TRS are synonymous. Essentially, all individuals who meet the criteria for TRS will also meet the CE criteria; however, we believe that using a CE framework more closely reflects the rationale for the study (to develop neurobiological indicators of an illness that would benefit from clozapine treatment). While related to early treatment non-response in EPP populations, measuring biomarkers between those with EPP who are clozapine-eligible versus treatment-responding presents a clinically useful framework that may allow EPP patients to be stratified to clozapine at an earlier time based on neurobiological markers. Furthermore, the term CE implies a more hopeful terminology than TRS. Because many individuals with TRS achieve a good response to clozapine, the term TRS, though used frequently, is a misnomer. However, definitions for TRS, and thus CE, have been rigorously studied. Accordingly, the current study compared glutamate levels in the ACC between individuals with EPP who were responders to first-line antipsychotic medications relative to individuals who were clozapine-eligible, based on criteria that are consistent with standard clinical practice (32). This was the first study to expand upon findings linking elevated glutamate with poor treatment response to clozapine eligibility. However, similar to previous EPP studies in this area, we hypothesized that clozapine-eligible individuals would have higher levels of glutamate in the ACC relative to treatment responders.

## Methods

### Participants

Individuals with EPP were recruited from the Nova Scotia Early Psychosis Program (NSEPP) in Halifax, Nova Scotia, Canada. All individuals being followed by NSEPP between January 2022 and August 2024 were eligible for participation. NSEPP, a specialized EPP team, provides multidisciplinary care for individuals experiencing a first episode of psychosis and has an active roster of approximately 250 young adults. Patients are followed clinically for a period of 5 years. General exclusion criteria for the study were as follows: having a primary diagnosis of a substance-induced psychotic disorder, having a history of significant head trauma with loss of consciousness for >30 min, inability to provide informed consent, unstable neurological or medical illness, or having any contraindications to undergo an MRI. This study was approved by the Research Ethics Board at Nova Scotia Health Authority (NSHA), file 1025560. All participants provided written informed consent prior to participation.

### Study design

Using a cross-sectional naturalistic design, individuals with EPP who were clozapine-eligible were compared with individuals who met criteria as treatment responders. To determine group status, participants met with a psychiatrist who completed an assessment of symptom severity using the Positive and Negative Symptom Scale (PANSS) (33), illness severity using the Clinical Global Impression-Schizophrenia (CGI-S) (34), and functioning with the Social and Occupational Functioning Assessment Scale (SOFAS) (35). DSM-5 diagnoses were established collaboratively through case discussions with members of the individual’s care team (psychiatrist, nurse case manager) using the best-estimate procedure for diagnosis (36). Previous medication trials were obtained by asking the participant, as well as reviewing the electronic medical record, and through discussion with the individual’s psychiatric care team. Assessments of medication adherence during trials were obtained through discussion with the participants, reviewing the electronic medical record, and through discussion with the care team when warranted. Criteria for clozapine eligibility was according to modified treatment response and resistance in psychosis working group consensus criteria (32) as follows: have undergone two trials of antipsychotic treatment for at least 6 weeks with at least 600 mg of chlorpromazine equivalents daily (37), illness severity rating of greater than or equal to 4 (moderate) on the CGI-S and greater than or equal to 4 (moderate) on 2 PANSS symptom items, or at least one symptom with a severe rating (despite treatment with adequate antipsychotic medication), moderate functional impairment as per SOFAs (<60), estimated medication compliance of 80% of prescribed doses during the 6-week treatment trials, and have not yet undergone a trial of clozapine. All the above criteria needed to be satisfied in determining clozapine eligibility. The designation of treatment responders required having ratings of no more than mild severity on all PANSS items sustained for a minimum of 12 weeks, with functional impairment rated as mild or better (60 or greater SOFAS score); currently receiving treatment with a single antipsychotic medication; and estimated medication compliance of 80% of prescribed doses during the 6-week treatment trials. Duration of untreated psychosis (DUP) was defined as the time in weeks from the first onset of any positive symptoms to initiation of treatment as per Norman and Malla (38). All participants were taking antipsychotic medications as prescribed by their care team.

### Sample size

A power analysis was conducted using G*power (Version 3.1) to approximate the desired sample size for the proposed study. Two papers examining glutamate levels and treatment response in an early psychosis sample were reviewed for this purpose (23, 24) (note, however, that neither specifically investigated clozapine eligibility, as this has not yet been studied). Egerton et al. (23) used a simple *t*-test to compare treatment responders with non-responders, with a resultant effect size *d* of 1.11 with an alpha set to 0.05. They used an *a priori* sample size analysis to produce an estimate of 19 per group. Similarly, the 2018 study by Egerton et al. (24) used a repeated measures design power analysis (using the reported main effects output partial n2 value of 0.11 with an f effect size of 0.3515), resulting in an estimated sample size of 21 per group for a three-group analysis (treatment responders, non-responders, and healthy controls). Given the consistency of these two analyses, we believe that a projected sample size of 21 individuals per group (clozapine-eligible and treatment responders) will allow for testing of our hypothesis.

### MR acquisition

Neuroimaging took place at the Biomedical Translational Imaging Center (BIOTIC) at Nova Scotia Health using a 3-Tesla GE Healthcare Model MR750 whole-body magnetic resonance scanner (GE Healthcare MR750, Milwaukee, WI, USA). To acquire brain images, a 3D T1-weighted inversion recovery fast spoiled gradient recalled (IR-FSPGR) sequence (slice thickness of 1 mm, isometric) in the sagittal plane (TE = 1.34 ms, TR = 4.056 ms, FOV = 256 mm, and flip angle = 9) was used. For the purposes of this study, glutamate was measured in the ACC as the previous literature supports this region as being intricately important in the pathophysiology of schizophrenia (24, 25).

**FIGURE 1:**
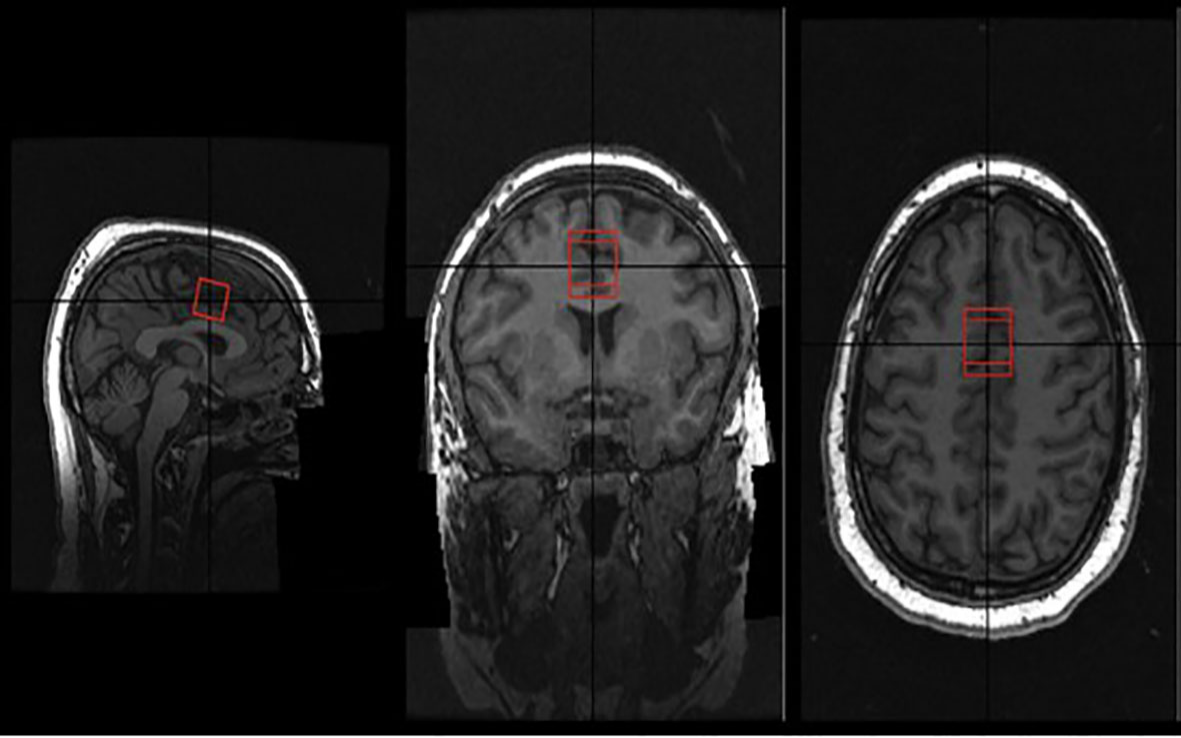
Voxel placement in ACC.

A 2.0-cm × 2.0-cm × 2.0-cm (8 cm^3^) ^1^H-MRS voxel was placed in the bilateral dorsal ACC (see **Figure 1**). The posterior end of the voxel was set to coincide with the precentral gyrus, and the caudal face of the voxel coincided with the most caudal location that is not part of the corpus callosum. Additionally, we had a coding script that allowed us to check the voxel placement after the scan to ensure our placements were as consistent as possible among the scans. The duration of the scan lasted approximately 30 min. We utilized the Point RESolved Spectroscopy (PRESS) sequence (TE = 40 ms; TR = 3,000 ms; 128 averages) in ^1^H-MRS acquisition (39). Previous studies have shown this PRESS sequence to be highly reliable in the repeated measurement of glutamate (39–41).

The quality criteria for spectral profiles was set at a linear combination of model (LCModel) signal-to-noise ratio (SNR) greater than 15, a scanner full-width at half maximum (FWHM) of the water peak less than 10 Hz, and a percent error (technical error) in the estimation of ^1^H-MRS concentration levels lower than 18% standard deviation/Cramer-Rao lower bounds (%SD) around the mean value for each specific signal as reported by the LCModel.

To account for potential scanner drift, we tested the ^1^H-MRS Spectroscopy phantom (GE Healthcare, Milwaukee, WI, USA) monthly over the course of the study (42). This testing allows us to evaluate quality assurance and avoid potential mistakes or defects made by the machine (42). The MRI scanner also underwent a software upgrade in November 2022. There were no significant changes in data quality parameters from pre- to post-scan (please see Supplementary data section).

**FIGURE 2:**
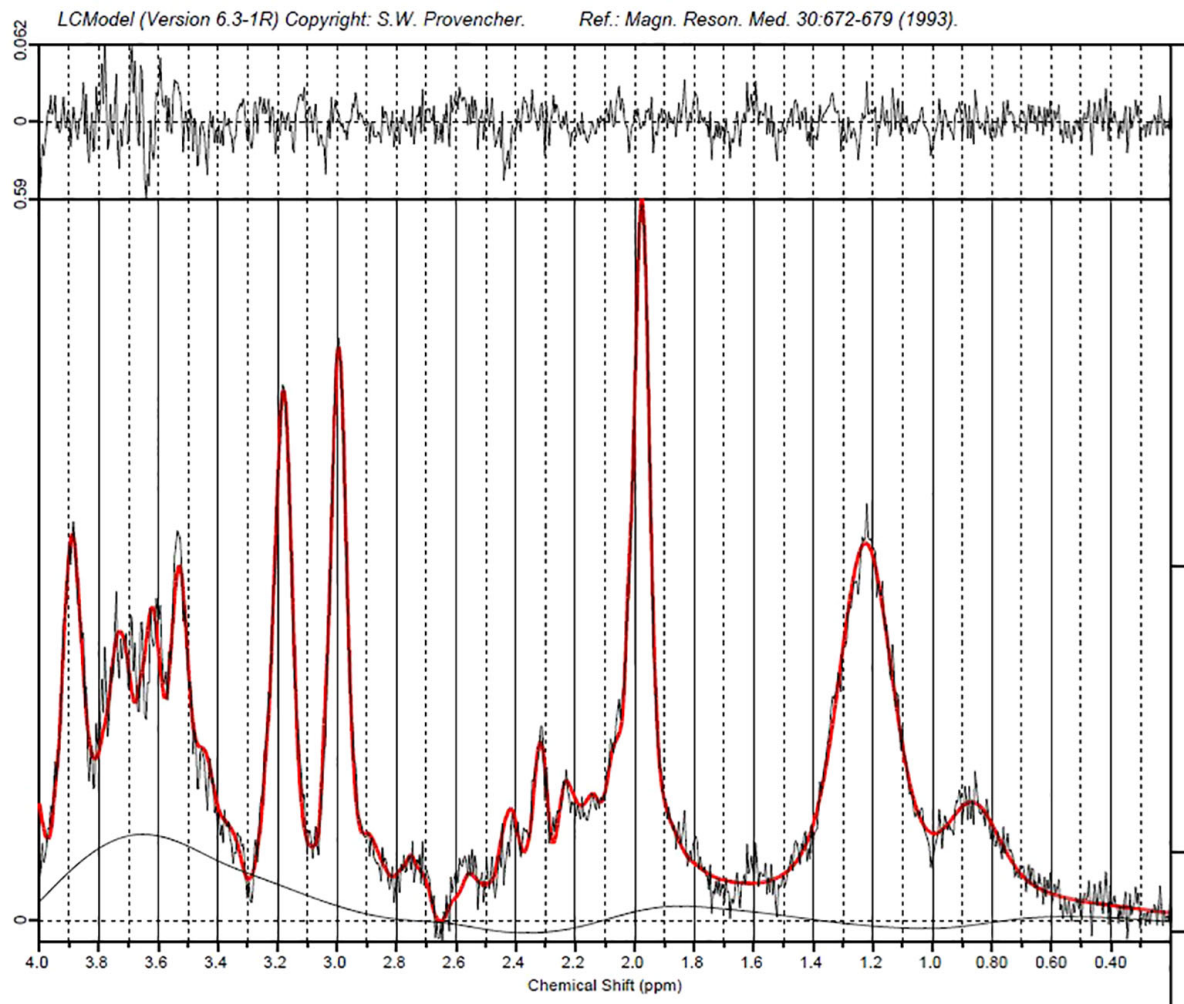
Example of fitted MRS spectrum from a single participant.

MRS data were fitted with LCModel (43). The LCModel is an automatic and objective measure. The program analyzes the spectra and produces a maximum likelihood estimate of metabolite concentrations as well as a %SD for each estimate (43). The unsuppressed water spectrum was used for internal referencing of glutamate concentration. The T1-weighted sequence was used for tissue type segmentation in each voxel of interest using Gannet and SPM12 (44, 45). (See **Figure 2** for an example of a fitted spectrum from a single participant).

The LCModel metabolite values were corrected for voxel tissue content using the formula reported by McQueen and colleagues (46): Mcorr = *M* × (wm + 1.21 × gm + 1. 55 × CSF)/(wm + gm), where *M* is the uncorrected metabolite, and wm, gm, and CSF represent the proportion of white and gray matter and cerebrospinal fluid content in the voxel. This formula assumes that CSF water concentration is 55,556 mol/m^3^ and the LCModel default brain water concentration is 35,880 mol/m^3^ (47, 48).

### Statistical analysis

All statistical tests were performed using R Statistical Software (v4.4.2) (49). Differences in demographical variables between groups were calculated using *t*-tests for continuous variables and chi-square analyses for dichotomous variables. Outliers were identified and excluded from the linear models *a priori* based on the interquartile range (IQR) method, where Q1 and Q3 are the first and third quartiles, and IQR is the interquartile range (Q3–Q1). Values above Q3 + 1.5 × IQR or below Q1 − 1.5 × IQR were considered outliers. Values above Q3 + 3 × IQR or below Q1 − 3 × IQR were considered extreme outliers. Linear models with a categorical (group) predictor were used to assess the differences between glutamate and glutamine. Clinical variables found to be significantly different between groups were systematically added to the linear model. A data-driven approach was used to select covariates to be added to the linear model; after identifying clinical variables as potential covariates, variables were added individually to a linear model, and comparisons between the base model (no covariates) and the updated model were performed using the flexplot package for R (v0.22.4) (50). This approach allowed us to select the model that best fit the data for each metabolite, based on objective measures of fit such as Akaike information criterion (AIC), Bayesian information criterion (BIC), Bayes factor, and *R*^2^. Model comparisons supported a base model with no covariates included in the model for comparing glutamate between groups. For comparing glutamine between groups, model comparison did not support the addition of most potential covariates. However, a model including the DUP as a covariate was supported by a higher *R*^2^ and adjusted *R*^2^, in comparison to the base model. Comparisons of AIC and BIC did not conclusively support the base model or the model with DUP. Considering the increased explanatory power of the model with DUP (*R*^2^) and clinical evidence linking DUP to TRS (2), the model with DUP was selected to compare group differences in glutamine.

## Results

In total, 51 individuals consented to participate in the study. Two individuals withdrew from the study during the MRI, two individuals had poor quality MRS data, and one individual was determined not to be eligible as they were experiencing an acute relapse of psychotic symptoms secondary to medication non-adherence. Therefore, the final sample consisted of 46 individuals, with 24 meeting criteria as clozapine-eligible and 22 as treatment responders.

All individuals were taking antipsychotic medication at the time of scanning, with 56.5% of the sample currently receiving long-acting injectable (LAI) antipsychotic treatment. Seventeen individuals were prescribed aripiprazole, 15 paliperidone, 6 olanzapine, 4 quetiapine, 2 risperidone, and 2 lurasidone. Group demographic and clinical data are summarized in **Table 1**. The groups did not differ in terms of age, sex, diagnosis, family history of psychosis, LAI use, and nicotine or cannabis use. The CE group had significantly higher PANSS scores, a longer DUP, and worse social functioning and were taking higher doses of antipsychotic medication (see **Table 1**).

**Table 1:** Sample demographics and clinical information per group.

Measures	TR (N = 22)	CE (N = 24)	t/χ^2^	P-value
Age (mean/SD)	25.32/3.55	25.88/5.67	−0.395	0.347
PANSS (mean/SD)				
Total	38.23/5.79	70.13/14.72	−9.506	<0.001*
Positive	8.27/1.39	18.67/4.29	−10.847	<0.001*
Negative	10.05/2.68	19.38/6.75	−6.05	<0.001*
General	19.91/4.19	32.79/7.54	−7.07	<0.001*
SOFAS (mean/SD)	68.18/15.29	38.00/14.38	6.89	<0.001*
CGI-S	2.67/0.57	5.05/0.94	−9.69	<0.001*
DUP (mean/SD)	11.41/12.35	25.88/14.64	−3.606	<0.001*
CPZ eq (mean/SD)	229.55/133.77	592.17/206.68	−7.00	<0.001*
Education (years) (mean/SD)	13.36/1.77	12.81/2.52	−0.395	0.347
Sex (male/female)	16/6	19/5	0.262	0.609
Diagnosis S/SA/SF/U	18/2/1/1	24/0/0/0	9.174	0.328
Nicotine Y/N	12/10	11/13	0.348	0.555
Cannabis Y/N	11/10	5/19	3.616	0.057
Fam Hx Y/N	6/15	10/14	1.951	0.377
LAI Y/N	12/10	14/10	0.067	0.796

### Spectral quality and control

Normality of data and equality of variances were assessed using Shapiro–Wilk and Levene’s tests, respectively. While FWHM and LCModel SNR were approximately normal in both groups, scanner SNR and line width each deviated from normality in at least one group. Based on these preliminary tests, Mann–Whitney *U* tests were used to compare scanner SNR and line width due to non-normality. Independent samples *t*-tests were used for FWHM and LCModel SNR, where assumptions of normality and equal variances were met. The mean scanner SNR did not differ significantly between treatment responders [mean (SD), 119.23 (± 26.61)] and clozapine-eligible individuals [113.41 (± 20.11), *z* = −0.01, *p* = 0.91]. Similarly, no difference was found in line width between treatment responders [6.07 (± 0.46)] and the clozapine-eligible group [6.07 (± 0.47), *z* = −0.03, *p* = 0.98]. Mean FWHM was also comparable across groups: *M* = 0.034, SD = 0.006 in responders; *M* = 0.035, SD = 0.007 in clozapine-eligible (*t* = −0.28, *p* = 0.78). Lastly, the LCModel SNR did not differ significantly: *M* = 26.38, SD = 6.80 in responders; *M* = 26.50, SD = 5.60 in clozapine-eligible (*t* = 0.06, *p* = 0.95). These findings indicate that MRS data quality metrics are consistent across both treatment response and clozapine-eligible groups.

### Differences in metabolite levels

**Figure 3:**
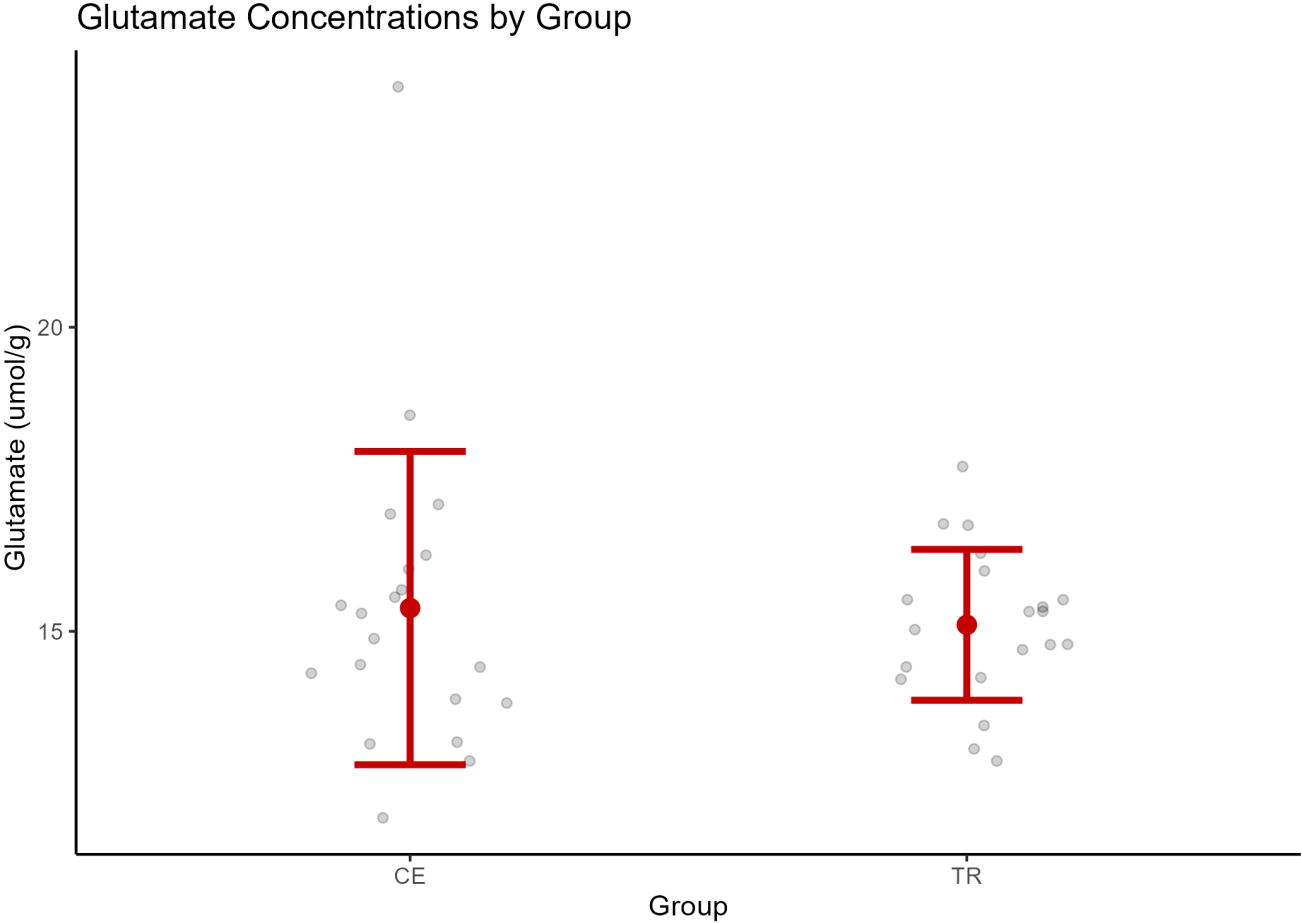
Glutamate concentrations by group (CE, clozapine-eligible; TR, treatment responders). The central point represents the mean, and vertical bars represent the standard deviation.

A one-way ANOVA was used to examine the difference in ACC glutamate levels between groups. See **Table 2**. Two cases were excluded prior to analysis due to outlier ACC glutamate values based on the aforementioned IQR method; however, this did not have a significant impact on the model results. ACC glutamate was not found to be significantly different between CE (*M* = 15.38, SD = 2.58) and TR (*M* = 15.11, SD = 1.24) individuals [*F* (1, 38) = 0.18, *p* = 0.67]. See **Figure 3**.

**Table 2:** Fixed-effects ANOVA results using ACC glutamate as the criterion.

Predictor	Sum of squares	*df*	Mean square	*F*	*p*	Partial *η*^2^	Partial *η*^2^ 90% CI [LL, UL]
(Intercept)	879.72	1	879.72	215.24	0.000		
Group	0.76	1	0.76	0.19	0.668	0.00	[0.00, 0.09]
Error	155.31	38	4.09				

LL and UL represent the lower limit and upper limit of the partial *η*^2^ confidence interval, respectively.

**FIGURE 4:**
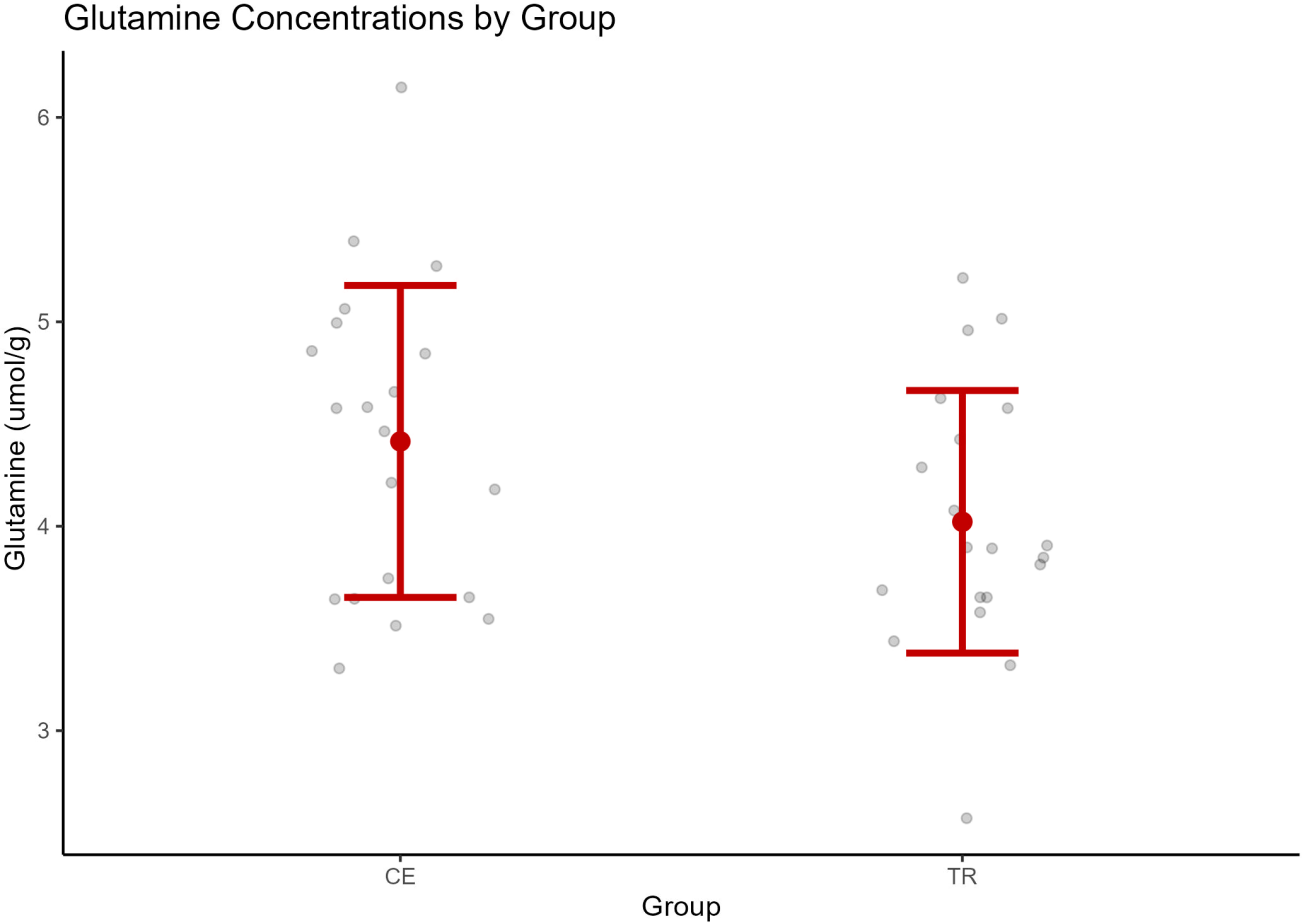
Glutamine concentrations by group (CE, clozapine-eligible; TR, treatment responders). The central point represents the mean, and vertical bars represent the standard deviation.

When comparing glutamine between groups, one case was excluded prior to analysis due to an extreme outlier glutamine value based on the IQR method described above. One-way ANCOVA was used to examine group differences in glutamine. See **Table 3**. Duration of untreated psychosis differed between the two groups, and model comparisons (see methods for details) supported adding DUP as a covariate in the model to examine group differences in glutamine. Considering DUP as a covariate in the model, glutamine was elevated in the CE (*M* = 4.41, SD = 0.76) group [*F* (1, 37) = 5.44*, p* = 0.043] relative to the TR group (*M* = 4.02, SD = 0.64). See **Figure 4**. Excluding the extreme case had a significant impact on the results of the model, such that the difference in glutamine between groups was not significant when the extreme case was included in a later model.

**Table 3:** Fixed-effects ANCOVA results using ACC glutamine as the criterion.

Predictor	Sum of squares	*df*	Mean square	*F*	*p*	Partial *η*^2^	Partial *η*^2^ 90% CI [LL, UL]
(Intercept)	51.82	1	51.82	105.01	0.000		
Group	2.17	1	2.17	4.39	0.043	0.11	[0.00, 0.27]
DUP	0.64	1	0.64	1.29	0.263	0.03	[0.00, 0.17]
Error	18.26	37	0.49				

LL and UL represent the lower limit and upper limit of the partial *η*^2^ confidence interval, respectively.

## Discussion

This was the first study to investigate differences in ACC glutamate in early-phase psychosis in individuals who were CE relative to those who responded to first-line dopamine-blocking antipsychotic medications. Contrary to our hypothesis, we did not find an association between elevated glutamate and treatment responsiveness status. However, we did observe significantly higher levels of glutamine, an amino acid precursor to glutamate, in the clozapine-eligible group after controlling for duration of untreated psychosis. Overall, these results support the theory of disrupted glutamatergic metabolism in treatment-resistant schizophrenia. Furthermore, these results contribute valuable information to our current understanding of the neurobiology underlying treatment resistance. Elevated glutamatergic metabolites appear to be associated with lack of response to antipsychotic medication and thus may be indicative of an overall phenotype that does not respond well to dopamine-blocking medications, satisfying the criteria for treatment-resistant schizophrenia longitudinally.

We found elevated levels of glutamine, but not glutamate in the CE group. In the glutamine/glutamate cycle, glutamine is released by astrocytes and then taken up by neurons to replenish stores of glutamate to be released (51). One possible explanation is that to prevent glutamate excitotoxicity, astrocytes may be overcompensating to avoid excess glutamate; however, prior review outside of schizophrenia or psychosis research has broadly suggested that malfunctioning glutamine homeostasis may mediate dysfunctional glutamate signaling and related excitotoxicity across multiple disorders (51), and considering previous spectroscopy findings, our finding supports this framework. Glutamine has previously been implicated in the pathophysiology of schizophrenia, such that glutamine was increased in the mPFC (52) and left ACC (53) of never treated patients compared to controls. Relatedly, glutamine was found to be lower in the ACC of medicated schizophrenia patients compared to healthy controls (54), implicating a potential impact of medication on glutamine. Conversely, more recent evidence has found elevated glutamine in the dorsal ACC of patients subject to long-term antipsychotic treatment and a positive association between glutamine and positive symptom severity (55). This finding supports the NMDA receptor hypofunction or glutamate model of schizophrenia (55) that is often associated with TRS (11); ketamine (an NMDA receptor antagonist) has been shown to increase glutamine in the ACC (56), while presynaptic glutamate inhibitors have prevented ketamine-induced psychotic symptoms (57). While we did not find elevated glutamate in the CE group, our finding of elevated glutamine is consistent with previous research that has suggested that increases in presynaptic glutamate release may lead to increased glutamine concentrations (55), and expands on these findings by demonstrating that this may be even more disturbed in TRS populations compared to treatment responders. In the context of the aforementioned glutamine literature and of the many previous studies to find elevated ACC glutamate in TRS populations, it may be the case that our finding of increased glutamine is an indicator of disrupted glutamine homeostasis in TRS that may lead to related glutamatergic excitotoxicity precipitating neuronal damage and dysconnectivity in frontal regions in TRS (22).

Beyond supporting the possibility of a glutamatergic subtype of schizophrenia, elevated glutamine may also be interpreted within emerging multimodal biomarker frameworks of psychosis. Elevated astrocytic glutamine levels have been linked to mitochondrial dysfunction in other medical conditions (58). Relatedly, a growing body of literature suggests a role of mitochondrial dysfunction in the pathophysiology of schizophrenia (59), and it has been suggested that the NMDA receptor and glutamate excitotoxicity have a role in mitochondrial processes (59, 60). It has also been suggested that mitochondrial dysfunction may disrupt the glymphatic system, potentially impairing the clearance of waste products in the brain (61). Recent preliminary evidence suggests that glymphatic system dysfunction may be present in early psychosis and during acute psychotic episodes, potentially contributing to altered metabolite clearance and glutamatergic imbalance (62, 63). Within this framework, glutamine elevation may reflect not only synaptic or metabolic dysregulation, but also broader system-level impairments relevant to biologically distinct subgroups of early psychosis. Integrating neurochemical markers with complementary modalities may therefore help refine biologically informed subtypes of early psychosis and inform the development of more targeted interventions.

Only a few studies have investigated glutamate and indices of treatment response cross-sectionally as in our study. Egerton and colleagues (23) found that ACC glutamate was elevated in individuals with first-episode psychosis who failed to obtain symptomatic remission after 6 months of antipsychotic medication treatment. Several groups subsequently found elevated baseline glutamate to be associated with measures of response at 1 month (Egerton et al., 2018), 6 weeks (25), and 3 months (26) of antipsychotic treatment. However, others have not found similar associations between ACC glutamate and response to treatment (27–29). While potentially related to a defined symptomatic reduction at a discrete interval, clozapine eligibility requires a lack of response longitudinally, having tried more than one antipsychotic medication, as well as some level of functional impairment. Defining treatment response more closely in keeping with clinical guidelines (31) did not find any differences in ACC Glx (glutamate + glutamine) at baseline in individuals who later failed to respond to two antipsychotic trials after 6 months. However, only a small proportion of the sample went on to meet criteria for treatment resistance longitudinally (*N* = 12). This is the only other study to characterize lack of response most consistent with guidelines for clozapine utilization. There are no other studies in EPP comparing glutamatergic metabolite levels cross-sectionally in a sample of individuals who currently meet criteria for CE or treatment responsiveness. Two meta-analyses in chronic schizophrenia did not find significant differences in ACC glutamate in TRS versus responders; however, many of the individuals in the TRS group were taking clozapine, which may be associated with a reduction in glutamate.

The strengths of this study include the focus on CE, a clinically relevant phenomena, with direct implications on patient care. The determination of CE was in keeping with clinical practice patterns and guidelines for when clozapine should be recommended and expands upon previous research that has characterized lack of response as failing to improve in symptoms at various discrete intervals. An additional strength is the representativeness of the sample; most individuals with early psychosis were eligible to participate in the study so long as they were taking antipsychotic medication. The high use of LAIs in our sample is an additional strength. In any study assessing medication response, pseudoresistance due to non-adherence is possible, and individuals categorized as non-responders may simply not be taking their medication as prescribed.

While this study provides novel insights into the pathophysiology underlying clozapine eligibility, there are a few limitations worth mentioning. First, while efforts were made to verify medication adherence, no serum antipsychotic levels or pill counts were performed. Therefore, it is possible that some individuals with residual symptoms may have not been taking their medications as prescribed or could have benefited from higher medication doses for response, resulting in pseudoresistance. This said, rates of LAI use within our sample were relatively high (56.5%), although, even in these individuals, some may have experienced a more robust improvement in symptoms with higher medication doses. In addition, individuals were treated with various non-clozapine antipsychotic agents as recommended by their primary clinician. Accordingly, it is possible that some medications may have had differential impacts on ACC glutamate. There are currently no studies comparing the effect of ACC glutamate of different antipsychotic medications. A recent study investigated acute changes in Glx (glutamate and glutamine ratio) in healthy volunteers with no history of mental illness who took amisulpride (a D2/D3 antagonist) versus aripiprazole (a D2 partial agonist) and placebo. After 1 week, healthy volunteers who took both amisulpride and aripiprazole showed increases in Glx relative to placebo; however, only the increase for aripiprazole was statistically significant. While this suggests that dopamine partial agonists may lead to an acute increase in Glx, it is possible that this trend does not translate to clinical samples and does not indicate whether there are differences in glutamatergic metabolites over the longer-term, such as in our study. In rats, acute antipsychotic administration has led to increases in medial prefrontal cortex glutamate (64), while longer-term antipsychotic administration has been associated with reductions in glutamatergic metabolites (65). However, mechanistically, dopamine partial agonists like aripiprazole will act as functional dopamine antagonists in areas of the brain where dopamine excess is present, such as the mesolimbic pathway (66). Therefore, given their mechanism of action on reducing psychotic symptoms in the mesolimbic pathway of the brain is similar, significant differences over time in glutamate in the ACC may not be present; however, we acknowledge that further studies on a similar population would be required to make definitive conclusions.

In addition, the rates of cannabis use in this current sample is relatively high, although the rates of regular users did not differ significantly between the two groups. Given the high rates of cannabis use in our region in general (67), not including individuals who use cannabis would have led to significant challenges with recruitment. Chronic cannabis use has been associated with decreased ACC glutamate relative to healthy non-users (68, 69). Furthermore, we can only comment on the association of glutamate in the dorsal ACC and did not capture metabolites in more ventral regions of the ACC. Finally, individuals who volunteer to participate in a neuroimaging study tend to be those who are experiencing less severe symptoms, making it difficult to capture a truly representative sample.

## Conclusions

This is the first study to examine the association between ACC glutamate and clozapine eligibility, a clinically relevant phenomenon, in an early psychosis sample. This work is complementary to previous work that has linked ACC glutamate to treatment response in EPP, and allows a better picture of the continuum from early treatment response issues to eventually meeting the threshold for CE. While we did not find differences in ACC glutamate between treatment responders and those who were clozapine-eligible, glutamine was higher in the CE group, in line with the theory of a glutamatergic subtype of illness, manifesting in a lack of response to dopamine-blocking medications. Future studies should explore whether there is an association between ACC glutamate and glutamine at the initial presentation of psychosis and clozapine eligibility longitudinally. Characterizing the neurobiology of clozapine eligibility would support the stratification of individuals who may be candidates for clozapine earlier in the illness trajectory.

## Data Availability

The datasets presented in this article are not readily available because they contain clinical information that could compromise the privacy of research participants. Requests to access the datasets should be directed to kara.dempster@nshealth.ca.
