# Supplementary material for: Elevated glutamine but not glutamate is associated with clozapine eligibility in an early psychosis sample
Source: Front Psychiatry. 2026 Feb 24;17:1762696. doi: 10.3389/fpsyt.2026.1762696 (PMC12971675; doi:10.3389/fpsyt.2026.1762696)
Supplement: Supplementary file 1 [file Table1.docx]

**Supplementary Materials**

1. MRS Quality Control

The 3T Tesla MRI scanner underwent a software upgrade between November 2022 and December 2022. To evaluate potential effects on spectral data quality, pre- and post- upgrade scans were performed on a research assistant using the 32-channel head coil employed in prior study sessions. No significant issues were identified in the spectral data following the upgrade. Signal-to-noise ratio (SNR) increased from 18 (pre-upgrade) to 36 (post-upgrade), and full width at half maximum (FWHM) values increased from 4.9 Hz (pre-upgrade) to 5.5 Hz (post-upgrade). Cramer-Rao lower bounds (CRLB) for both glutamate and glutamine dropped following the upgrade, with glutamate going from 6% to 4%, and glutamine going from 26% to 12%, respectively. The combination of glutamate and glutamine, Glx, saw CRLB stay consistent across the upgrade, remaining at 5%. Upon completion of the upgrade, the same 32-channel head coil was used for all subsequent acquisitions to maintain consistency. In total, 26 participants were scanned prior to the upgrade, and 20 were scanned after the upgrade.
